# Effects of Glutathione on Mechanical Allodynia and Central Sensitization in Chronic Postischemic Pain Rats

**DOI:** 10.1155/2017/7394626

**Published:** 2017-10-25

**Authors:** Jinseok Yeo, Hoon Jung, Hyerim Lee

**Affiliations:** Department of Anesthesiology and Pain Medicine, Chilgok Hospital, School of Medicine, Kyungpook National University, Daegu, Republic of Korea

## Abstract

**Background:**

The chronic postischemia pain (CPIP) model is an animal model using ischemia/reperfusion injury that mimics the symptoms of complex regional pain syndrome type I. Glutathione (GSH) prevents ischemia/reperfusion injury by scavenging free radicals. We conducted this study to investigate the protective effect of GSH in CPIP rats via changes of mechanical allodynia and phospholyration of the N-methyl-D-aspartate receptor subunit GluN1.

**Methods:**

We divided 45 rats into 5 groups: sham, CPIP, CPIP + GSH 100 mg/kg, CPIP + GSH 200 mg/kg, and CPIP + GSH 500 mg/kg. Rats in the sham and CPIP groups received normal saline and rats in the other groups received GSH at the designated doses thirty minutes prior to reperfusion. Withdrawal thresholds were evaluated before sugery as well as 1, 3, and 7 days after surgery. pGluN1 level in the spinal cord was also measured.

**Results:**

GSH treated rats show a significant increase in the withdrawal thresholds of both hind paws as compared with the CPIP group dose-dependently. The expression of pGluN1 in the GSH treated rats significantly decreased as compared to the CPIP group (all* P* < 0.05).

**Conclusion:**

These findings suggest that GSH inhibited the development of mechanical allodynia and central sensitization in CPIP rats.

## 1. Introduction

Complex regional pain syndrome type I (CRPS I) is a devastating condition that usually affects a limb and is not accompanied by a clinically verifiable nerve injury [1]. CRPS I commonly develops following sprains, arthroscopic surgery, overly tight casting, and edematous soft tissue injuries. Many different mechanisms of action for the development of CRPS I have been suggested. These include an inflammatory process, centrally mediated sympathetic stimulation, central sensitization of pain, an autoimmune process, neurogenic inflammation, and ischemia-reperfusion injury [2].

The chronic postischemia pain (CPIP) is an animal model that mimics the symptoms of CRPS I [3]. In this model, a hind paw ischemia-reperfusion (IR) injury produces persistent mechanical and cold allodynia, edema, and microvascular dysfunction in rat hind paw without evidence of accompanying nerve damage. IR injury occurs when the blood supply returns to a tissue following a period of insufficient blood flow. Prolonged ischemia results in the accumulation of nitric oxide (NO) and hypoxanthine in the affected tissue [4]. The reintroduction of molecular O_2_ to ischemic tissue upon reperfusion results in the production of superoxide (O_2_^−^) and xanthine through the action of xanthine oxygenase [5]. The reaction of NO with O_2_^−^ results in the production of peroxynitrite (ONOO^−^) [6]. ONOO^−^ is a strong oxidant that exerts its toxic effects through lipid peroxidation, mitochondrial damage, protein nitration and oxidation, depletion of glutathione (GSH) reserve, and DNA damage [7]. The formation of ONOO^−^ in the ischemic tissue leads to vascular dysfunction and tissue damage [8]. ONOO^−^ is also known to be involved in such physiological processes as inflammation, nerve injury, opioid induced hyperalgesia, and IR injury [9, 10]. In CPIP rats, ONOO^−^ is involved in development of allodynia and N-methyl-D-aspartate (NMDA) receptor mediated central sensitization [11].

GSH is the most abundant antioxidant in the cell. Ischemia-reperfusion injury depletes GSH in the tissue and supplementation with GSH has been found to reduce associated tissue damage [12, 13]. GSH has also been found to reduce ONOO^−^ associated injury [14]. However, no previous studies have evaluated the effect of GSH on the development of mechanical allodynia following IR injury. We hypothesized that the protective effect of GSH on ONOO^−^ associated injury could attenuate the development of mechanical allodynia and central sensitization in CPIP rats that mimics the symptoms of human CRPS I. Therefore, we conducted this study to investigate the protective effect of GSH in CPIP rats via changes in withdrawal threshold following mechanical stimulation of a hind limb and in the spinal activation of the N-methyl-D-aspartate (NMDA) receptor subunit GluN1 (pGluN1).

## 2. Materials and Methods

### 2.1. Animals

Forty-five male Sprague-Dawley rats (320–340 g) were used in this study (Koatech, Gyeonggi-do, Korea). All rats were housed at the animal care facility under specific pathogen-free conditions. They were allowed free access to food and water and were exposed to a light-and-dark cycle of 12 hours each. Care was provided according to National Institutes of Health guidelines on laboratory animal welfare. The rats acclimated to their cages for 7 days prior to the start of the experiment.

### 2.2. Experimental Protocol

**Figure 1:**
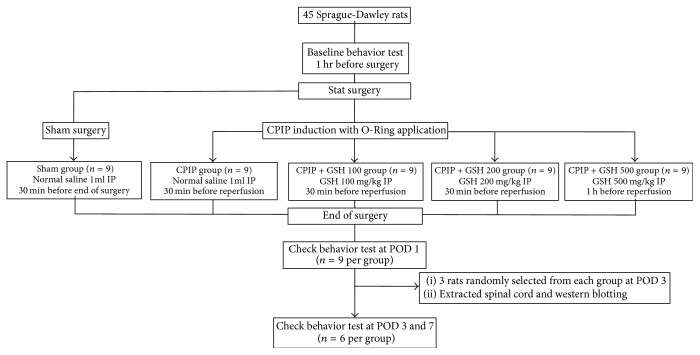
Flow of the study. CPIP: chronic postischemia pain, GSH: glutathione, IP: intraperitoneal injection, and POD: postoperative day.

The Institutional Animal Care and Use Committee at Kyungpook National University approved the experimental protocol. A total of 45 male rats were evenly distributed between five groups, with nine rats per group: (1) sham group, (2) CPIP group, (3) CPIP + GSH 100 mg/kg group, (4) CPIP + GSH 200 mg/kg group, and (5) CPIP + GSH 500 mg/kg group. The rats in sham group underwent a sham procedure, while the rats in the other groups underwent the CPIP procedure. Rats in the sham group and the CPIP group received intraperitoneal injections of 1 cc of normal saline 30 minutes prior to reperfusion. Rats in the CPIP + GSH 100 mg/kg group, CPIP + GSH 200 mg/kg group, and CPIP + GSH 500 mg/kg group received intraperitoneal injections of the designated dose of GSH mixed with 1 cc of normal saline 30 minutes prior to reperfusion (Figure 1).

### 2.3. Hind Paw Ischemia and Reperfusion

**Figure 2:**
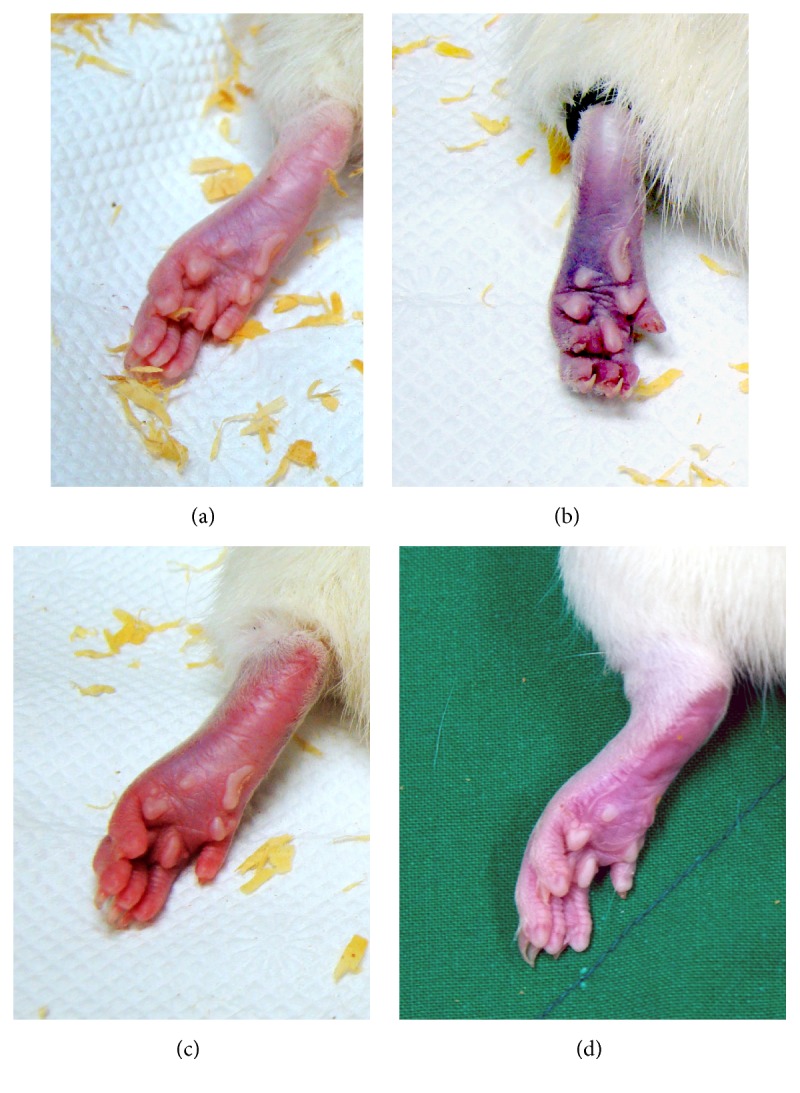
Photographs of the left hind paw in chronic postischemic pain rats. (a) Normal hind paw. (b) Hind paw during ischemic event: a tight-fitting O-Ring has been placed just proximal to the ankle joint; the paw shows cyanosis. (c) Hind paw 10 minutes after reperfusion: the paw shows swelling and hyperemia. (d) Hind paw 3 days after reperfusion: swelling and hyperemia are decreased; the paw appears dry and shiny.

The CPIP procedure was performed as described by Coderre et al. [3]. Anesthesia was induced in all rats with an intraperitoneal injection of 50 mg/kg of sodium phenobarbital. Anesthesia was maintained with an infusion of sodium phenobarbital at 20 mg/kg/hr. Once anesthetized, a Nitrile 70 Durometer O-Ring (O-Rings West, Seattle, WA) with a 5.5 mm internal diameter was placed around the left hind limb just proximal to the medial malleolus of each rat in the CPIP groups. Three hours after placement of the O-Ring, it was removed and reperfusion was allowed to occur. An O-Ring was placed identically on the rats in the sham group, but the device was cut so that it surrounded the ankle loosely and did not occlude blood flow to the paw. All rats recovered fully within 30 minutes of O-Ring removal (Figure 2).

### 2.4. Hind Paw Mechanical Allodynia

To allow acclimation to the environment, the rats were placed individually in a testing cage (23 × 16 × 24 cm^3^) with a mesh metal floor for 1 hour daily beginning 2 days before the start of the experiment. Thirty minutes before the experiment began the rats were again allowed to acclimate. The ipsilateral hind limb was then evaluated for mechanical allodynia. The mechanical threshold for allodynia was assessed by measuring the 50% withdrawal response to von Frey filaments (Stoelting, Wood Dale, IL, USA) using a modification of the up/down method [15]. Von Frey filaments were applied to the center of the plantar surface of the hind paw for 10 seconds or less if the rat responded to the stimulus by withdrawing, flicking, stamping, or licking its paw. The experiment was started with a filament producing a force of 2 g and was continued in either ascending (after a negative response) or descending (after a positive response) order. The minimum stimulus intensity utilized was 0.25 g and the maximum was 15 g. A 50% response threshold (g) was calculated from the response pattern and the force produced by the final filament. Both hind limbs were tested. A baseline recording of the withdrawal threshold was obtained 1 hour before infliction of the IR injury. On the first, third, and seventh days after CPIP injury, we evaluated the withdrawal threshold to determine the effects of GSH. All testing was performed by a researcher blinded to the treatments.

### 2.5. Western Blot Analysis

Three days after the procedure, three rats were randomly selected from each group. All rats were fully anesthetized and then rapidly sacrificed. The L4-5 spinal cord segment was harvested from each rat and then divided into a left and right side. The spinal cord sections were placed immediately in liquid nitrogen. The left side of the spinal cord sample was homogenized in 100 *μ*l of lysis buffer (20 mM Tris-HCl pH 8.0, 150 mM NaCl, 1 mM EDTA, 2 mM Na_3_VO_4_, 0.5 mM DTT, 10% glycerol, and 1% Nonidet P-40) plus a protease inhibitor cocktail tablet (Roche Diagnostics, Mannheim, Germany). The lysates were centrifuged at 13,000 ×g for 25 min at 4°C. The supernatants were collected and the proteins were quantified using the Bradford method (Protein Assay Kit I, Bio-Rad, Hercules, Ca, USA). Protein samples (40 *μ*g) were dissolved in a buffer solution (0.1 M Tris-HCl, 10% glycerol, 2% SDS, and 0.1% bromophenol blue). The protein samples were heated at 100°C for 5 minutes and then loaded into 10% SDS-polyacrylamide gels. After separation by SDS-polyacrylamide gel electrophoresis, the proteins were transferred to a nitrocellulose membrane and blocked with Tris-buffered saline (50 mM Tris-HCl pH 7.4 and 10 mM NaCl) in 3% skim milk at room temperature for 1 hour. The blots were incubated with anti-phospho-NR1 antibody (1 : 500, Ser897, ABN99, Millipore, Billerica, MA, USA) and anti *β*-actin antibody (1 : 5000, *β*-actin, sc-47778, Santa Cruz Biotechnology, Dallas, TX, USA) at 4°C overnight. The blots were washed with washing buffer (50 mM Tris pH 7.4 and 10 mM NaCl) and then incubated with anti-mouse horseradish peroxidase conjugated secondary antibodies (1 : 2000, anti-mouse IgG-HRP, 7076S, Cell Signaling Technology, Danvers, MA, USA) for 1 hour at 21°C. The blots were developed with an enhanced chemiluminescence kit (Amersham Pharmacia Biotech, Amersham, UK). *β*-Actin was used as an internal control. The densities of the protein blots were quantified using Image J 1.50i software (National Institutes of Health, Bethesda, MD, USA).

### 2.6. Statistical Analyses

The measured data are presented as the mean ± standard error of the mean (SEM) and were analyzed using the statistical program SPSS statistics for Windows, version 21.0 (SPSS, IBM Corp., Armonk, NY, USA.). Analysis of change over time for multiple groups was performed using a two-way ANOVA with one repeated measure (time) followed by the Tukey test. Differences in values between multiple groups were tested using the one-way ANOVA (when comparing more than two groups) followed by the Tukey test. Change over time in a single group was tested using the one-way repeated measures ANOVA. *P* < 0.05 indicated statistical significance.

## 3. Results

### 3.1. Hind Paw Mechanical Allodynia

**Figure 3:**
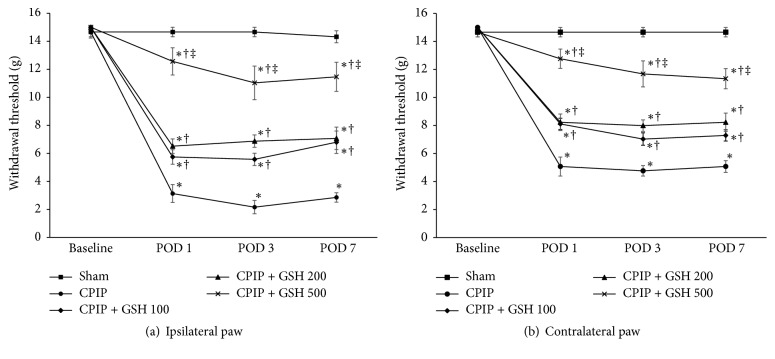
Withdrawal threshold in CPIP and sham rats. Rats in the sham and CPIP groups received saline. Rats in the CPIP + GSH 100, CPIP + GSH 200, and CPIP + GSH 500 groups received GSH 100 mg/kg, 200 mg/kg, and 500 mg/kg, respectively. CPIP: chronic postischemia pain; GSH: glutathione. ^*∗*^*P* < 0.05, repeated measurements ANOVA followed by the Tukey test compared to the sham group. ^†^*P* < 0.05, repeated measurement ANOVA followed by the Tukey test compared to the CPIP group. ^‡^*P* < 0.05, repeated measurement ANOVA followed by the Tukey test compared to the CPIP + GSH 100 group.

The withdrawal thresholds for both hind paws at baseline did not differ between the groups. There were no significant changes in the withdrawal thresholds of the ipsilateral and contralateral hind paws in the sham group during the entire study period. GSH treatment 30 minutes before reperfusion was associated with a significant increase in the withdrawal thresholds of both hind paws following reperfusion. The withdrawal thresholds of both hind paws in the CPIP and CPIP + GSH in the dose of 100 mg/kg, 200 mg/kg, and GSH 500 mg/kg groups on the first, third, and seventh days following reperfusion were significantly reduced as compared to the sham group at these same time points (*P* < 0.05). The CPIP + GSH 500 mg/kg group showed a significant increase in the withdrawal threshold as compared to the CPIP + GSH 100 mg/kg group (*P* < 0.05) in both hind paws at first, third, and seventh days after reperfusion (Figure 3).

### 3.2. Measurement of pNR1 in the Spinal Cord

**Figure 4:**
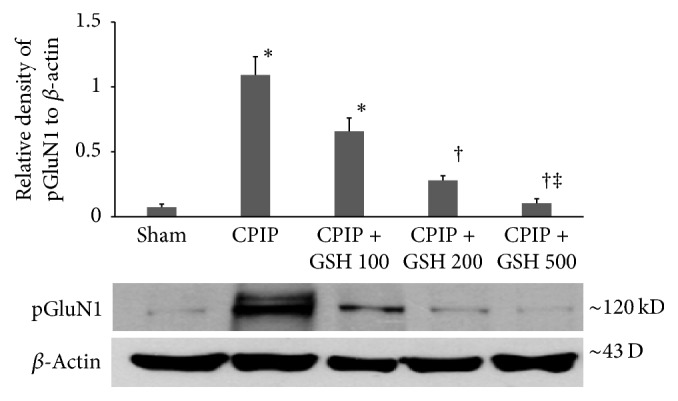
Changes in the relative density of pGluN1 protein in the ipsilateral (left) spinal cord segment (L4-5). Expression of pGluN1 was measured by western blot on post-op day 3. Three rats were randomly selected from each group and sacrificed. Data are expressed as mean ± standard error of the mean. *∗* indicates values significantly different from the sham group. † indicates values significantly different from the CPIP group. ‡ indicates values significantly different from the CPIP + GSH 100 group. *P* < 0.05 was considered statistically significant. CPIP: chronic postischemic pain, GSH: glutathione, and pGluN1: phosphorylated N-methyl-D-aspartate receptor subunit 1.

The relative densities of pGluN1 in the CPIP and CPIP + GSH 100 mg/kg groups were significantly higher than that in the sham group. The relative densities of pGluN1 in the CPIP + GSH 200 mg/kg and CPIP + GSH 500 mg/kg groups were significantly lower than that in the CPIP group. The relative density of pGluN1 in the CPIP + GSH 500 mg/kg group was significantly lower than that in the CPIP + GSH 100 mg/kg group (Figure 4).

## 4. Discussion

Our study has showed that the treatment with GSH prior to reperfusion attenuated mechanical allodynia and suppressed spinal phosphorylation of GluN1 in CPIP rats dose-dependently. These findings suggest that the development of allodynia in the CPIP rats results from central sensitization secondary to GluN1 phosphorylation, and treatment with GSH suppressed sensitization in a dose-dependent manner.

The proposed pathogenesis of CRPS I is microvascular injury that results from a persistent deep tissue ischemia and subsequent inflammatory reaction after reperfusion [16]. Microvascular injury in the affected limb activates peripheral nociceptors and initiates and maintains NMDA mediated central sensitization [17]. The CPIP model involves complete occlusion of blood flow to the hind paw and results in prolonged reactive hyperemia and edema following reperfusion. The rats in this model develop cold allodynia, mechanical hyperalgesia, and vascular abnormalities without evidence of nerve injury, which is similar to the findings in CRPS I patients [18]. Prolonged ischemia in CPIP rats results in the accumulation of oxidases. Accumulated xanthine oxidase is responsible for the overproduction of superoxide during reperfusion. Activation of nitric oxide synthase during ischemia results in the production of nitric oxide (NO) from vascular endothelium and neutrophils [19]. Superoxide is rapidly removed by superoxide dismutase (SOD), and NO diffuses rapidly into red blood cells [6]. The proximity of NO and superoxide in the vascular space may promote the generation of ONOO^−^ during early reperfusion [20].

ONOO^−^ is an extremely short-lived cytotoxic oxidant. It has a half-life of 1.9 seconds at pH 7.4, which is even shorter in vivo [21]. In spite of its short half-life, ONOO^−^ is able to diffuse through several cells and induce lipid peroxidation and DNA and protein damage [22]. ONOO^−^ damages capillary endothelial cells and arterial smooth muscle cells following IR injury. Injured endothelial cells swell and occlude the capillary lumen. Capillary occlusion and reperfusion of the tissue produce IR injury [23]. Endothelial cell dysfunction decreases NO production and results in arterial vasospasm [18, 24]. Microvascular dysfunction following IR injury results in ischemia of the paw muscle, and allodynia has been correlated with muscle ischemia in CPIP rats [25]. ONOO^−^ reportedly contributes to the development of chronic pain, and peripheral administration of ONOO^−^ has been shown to induce inflammatory hyperalgesia [9]. Peripheral nerve injury results in the production of ONOO^−^, and scavenging of ONOO^−^ has been shown to alleviate hyperalgesia and nerve degeneration [10]. Pretreatment of CPIP rats with an ONOO^−^ decomposition catalyst has been shown to decrease allodynia and prevent central sensitization by attenuating NMDA receptor activation [11].

GSH is an intracellular tripeptide thiol. It is a major water-soluble antioxidant and plays a central role in the modulation of nitric oxide [9, 26]. GSH converts ONOO^−^ to less harmful by-products such as S-nitrosoglutathione [27]. In the presence of ONOO^−^, GSH (1) relaxes vessel walls, (2) stimulates the activity of guanylate cyclase in endothelial cells, (3) attenuates ONOO^−^ induced hemolysis, and (4) inhibits platelet aggregation [14]. These actions by GSH may reduce endothelial cell damage and improve microcirculation. Exogenously administered GSH is reported to increase intracellular GSH levels, and infusion of GSH has been shown to decrease pain and improve microcirculation in patients with peripheral artery disease [28]. In our study, GSH injection 30 minutes prior to reperfusion resulted in an increase in the withdrawal threshold and suppression of pGluN1 expression in a dose-dependent manner. These results suggest that GSH decreased pain and central sensitization after IR injury by decreasing ONOO^−^ induced toxicity in CPIP rats.

In this study, we did not evaluate the dose-dependent effect of GSH on muscle ischemia and microvascular injury. However, IR injury is known to reduce the concentration of GSH in skeletal muscle [29], and the extent of hind paw muscle ischemia following CPIP injury is correlated with the development of mechanical allodynia [25]. In this study, supplementation with GSH prior to reperfusion suppressed the development of allodynia. This result suggests that GSH might attenuate pain by reducing microvascular damage after IR injury.

In conclusion, the results of the present study indicate that administration of GSH prior to reperfusion alleviates mechanical allodynia and spinal phosphorylation of GluN1 in CPIP rats. Our findings suggest that GSH counteracted microvascular dysfunction following IR injury. Further studies are needed to gain significant translatability by evaluating the antinociceptive potential of GSH analogs in ongoing clinical trials in the CPIP rats and CRPS I patients.
